# Assessing the impacts of climate change on climatic extremes in the Congo River Basin

**DOI:** 10.1007/s10584-022-03326-x

**Published:** 2022-02-28

**Authors:** Sara Karam, Ousmane Seidou, Nidhi Nagabhatla, Duminda Perera, Raphael M. Tshimanga

**Affiliations:** 1 Faculty of Engineering, University of Ottawa, Ottawa, ON Canada; 2 United Nations University, Institute for Water, Environment and Health, Hamilton, ON Canada; 3 School of Earth, Environment and Society, McMaster University, Hamilton, ON Canada; 4 United Nations University, Institute On Comparative Regional Integration Studies (UNU-CRIS), Bruges, Belgium; 5 Congo Basin Water Resources Research Center – CRREBaC, and Department of Natural Resources Management, University of Kinshasa, Kinshasa, DR Congo

**Keywords:** Congo River Basin, Climate indices, Droughts, Floods, Precipitation, Evapotranspiration, Downscaling, RCM, SPEI, SPI

## Abstract

The Congo River Basin, located in central Africa, is the second-largest river basin in the world, after the Amazon. It has a drainage area of approximately 3.7 M km^2^ and is home to 75 million people. A significant part of the population is exposed to recurrent floods and droughts, and climate change is likely to worsen these events. Climate change studies of the Congo River basin have so far focused on annual and seasonal precipitation, but little attention was paid to extreme climatic events. This study aims to assess future changes in rainfall-induced flash floods and drought regimes in the Congo basin from the present day to 2100, using four selected extreme climatic indices as proxies to these two natural disasters. The indices are the total annual precipitation (PCPTOT), the number of days where rainfall is above 20 mm (PCP20), the standardized precipitation index (SPI), and the standardized precipitation-evapotranspiration index (SPEI). The indices were calculated with the statistically downscaled output of eleven Regional Climate Models (RCMs) from the Coordinated Downscaling Experiment (CORDEX-AFRICA) under two Representative Concentration Pathways: RCP 8.5 (high emissions scenario) and RCP 4.5 (moderate emissions scenario). Precipitation and temperature simulated by the RCMs were statistically downscaled using quantile mapping, while wind speed, solar radiation, and relative humidity were projected using K-nearest neighbor downscaling. The evolution of the indices was then assessed between the reference period (1976–2005) and three future periods (2011–2040, 2041–2070, and 2071–2100). Multimodel average results suggest that (i) independently of the scenario and period, PCPTOT and SPI will increase in the north, east, and western extremities of the basin and decrease in the basin’s center. (ii) The maximum increase (+ 24%) and decrease (− 6%) in PCPTOT were both projected under RCP 8.5 in the 2071–2100 period. (iii) PCP20 will increase independently of the period and scenario. Under RCP 8.5, in the 2071–2100 period, PCP20 will increase by 94% on average over the whole watershed. (iv) The SPEI results suggest that in all periods and scenarios, the rise in evapotranspiration due to higher temperatures will offset annual precipitation increases in the north, east, and western extremities of the basin. Increased evaporation will exacerbate the decrease in annual precipitation in the center, leading to increased drought frequency in the entire basin.

**Supplementary Information:**

The online version contains supplementary material available at 10.1007/s10584-022-03326-x.

## Introduction

**Fig. 1:**
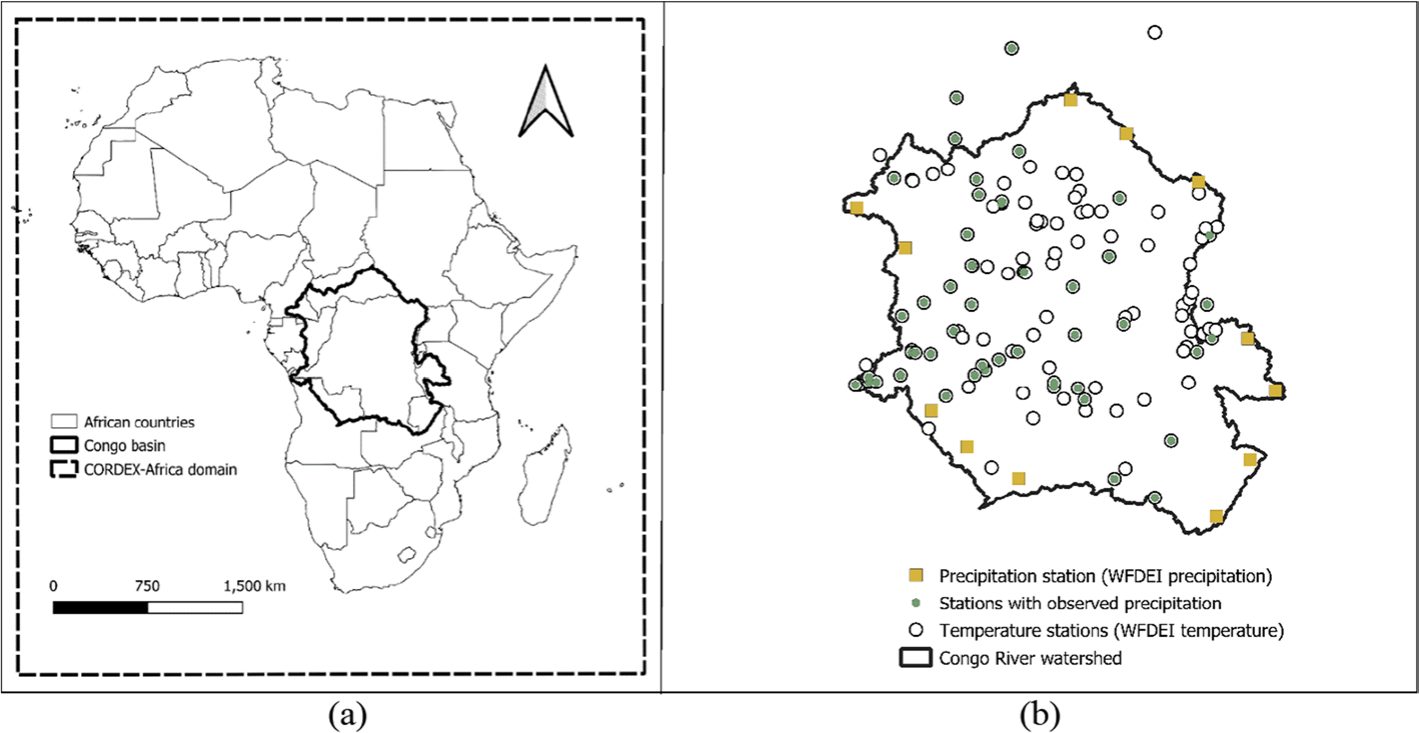
(**a**) Extent and location of the Congo basin. (**b**) Observation stations in (or near) the Congo basin for which precipitation and temperature data were available (either from direct observations or WFDEI reanalysis).

The Congo River Basin (Fig. 1) is in central Africa and has a drainage area of approximately 3.7 M km^2^. It is the second-largest river basin in the world, after the Amazon. It encompasses nine political boundaries, including Angola, Burundi, Central African Republic, Democratic Republic of Congo, Cameroon, Republic of Congo, Rwanda, Tanzania, and Zambia (Tshimanga et al. 2020). The Congo basin hosts the world’s second-largest rainforest, which is crucial for the global carbon cycle. Like other rainforests in the world, its distribution and composition are likely to be influenced by changes in regional rainfall characteristics resulting from global warming (Malhi and Wright 2004). The Congo basin, along with the western Pacific Ocean and the Amazon basin, is a significant source of major storms. It also experiences droughts: there is evidence of rainforest contraction and forest composition changes during dry periods in the past 3000 years (Lewis et al. 2009; Malhi et al. 2013). It is, therefore, vital that climatic changes in the basin be studied to provide credible information for mitigation and adaptation planning (Creese et al. 2019). Despite its importance, the basin remains primarily understudied and has not received adequate attention in hydrological and climate research (Tshimanga and Hughes 2014). Between 1960 and 2019, over 11,566,029 people were affected by flooding in the countries that comprise the Congo basin. There were 3062 casualties, and the total economic damages are estimated to be 96 billion USD (EM-DAT 2020). These extreme events in the Congo basin are related to rainfall variability, which may be exacerbated by climate change at a level that may challenge the livelihood of people living in the basin. Hence, it is crucial to analyze the current and future evolution of extreme events, such as precipitation and drought frequency/intensity, for the preservation of local infrastructure and livelihood sustainability, between the reference period (1976–2005) and three future periods (2011–2040, 2041–2070, and 2071–2100).

Several authors have examined the impacts of climate change on hydroclimatic variables in various regions, including the Congo basin. Tshimanga and Hughes (2014) used Climate Model Intercomparison Project phase 3 (CMIP3) GCMs to study the effects of climate change on the Congo basin's hydrology in terms of runoff in near-future projections of the northern sub-basins of the Oubangui and Sangha Rivers. Their results point to a significant increase in evapotranspiration and a 10% decrease in total runoff. Onyutha (2020) derived twelve Extreme Rainfall Indices (ERIs) from observed 1961–1990 daily rainfall across East Africa. The ERIs were calculated with the outputs of six Regional Climate Models (RCMs) driven by twenty-six CMIP5 (Climate Model Intercomparison Project phase 5) models. They found that the ensemble means of the RCMs reproduced observed ERIs better than the individual RCMs. Jenkins et al. (2002) examined the ability of GCMs to produce realistic climatology in West Africa using the Tropical Rain Measuring Mission (TRMM) satellite and simulated rainfall rates from the Community Climate Model version 3.6 (CCM3). Although GCMs do carry uncertainties, the authors stated that they offer a good solution to predicting climate change in West Africa due to their capability of capturing mesoscale systems and their accurate consideration of orographic features. Creese et al. (2019) conducted a study on climate change in the Congo basin, focusing on wetting in the December-February season (DJF), using nineteen CMIP5 GCM models. They were able to quantify and define the spectrum of rainfall variation in the basin between 1979–2005 and 2074–2100. They found that in DJF, the seasonal rainfall would vary from 2 to 160 mm throughout the various models between the past and future periods. Mao et al. (2019) analyzed river flooding in the basin in relation to what season the flooding occurred, to differentiate the different causes of flooding, such as intense rainfall events or an excess of soil moisture. They found a link between progressive climate change and an observed increase in inundation events throughout multiple watersheds globally, including the Congo basin. They also used the annual maximum daily flood extent to define a flood seasonality index. The mean flood date (MD) between 1953 and 2004 and the seasonality index were used to qualitatively classify floods in the Congo basin (among other watersheds) into several event-driven categories. They showed that the combination of soil moisture excess and extreme precipitation events was the main contributor to flood events in the Congo basin. Ndehedehe et al. (2019) modeled the impacts of global multi-scale climatic drivers on mean annual hydroclimatic extremes such as the Standardized Precipitation Index (SPI) and Standardized Runoff Index (SRI) between 1901 and 2014 in the Congo basin. Statistical relationships and coupled variability between SPI and global climate models were examined. They developed a predictive framework with a non-linear autoregressive neural network to analyze the connectivity between SPI patterns and global ocean–atmosphere regimes. The study showed that the Congo basin could be characterized by severe multi-year droughts between 1901 and 2014, which affected 40–50% of the land area. The study showed that as of 1994, the hydrological regimes of the basin have changed due to drought intensification.

The focus of the above studies that considered the Congo basin were either extreme events in the recent past (Ndehedehe et al. (2019); Mao et al. (2019)) or average annual and seasonal precipitation and evapotranspiration in the future (Creese et al. (2019)). The only study dealing with extreme events in the future (Onyutha 2020) focused on east Africa and only considered rainfall-related indices. Furthermore, the RCM data they used was not further corrected, while it is known that RCM precipitation can significantly differ from ground observations, in terms of both magnitude and seasonal patterns. Given the importance of the entire Congo basin and the vulnerability of its inhabitants, up-to-date knowledge about flood and drought regimes in the current and future periods has become increasingly relevant. Annual average precipitation and evaporation examined in previous studies are pertinent for practical use, but extreme values have a greater impact on the vulnerability of the basin’s population. Understanding climatic extremes can help improve water resources management and water-related disaster risk reduction and guide adaptation policies. Therefore, the current study aims at assessing future changes in rainfall and drought regimes in the Congo basin from the present time to 2100, using four selected climate indices: the average annual total precipitation, PCPTOT; the number of days where precipitation is above 20 mm, PCP20; The Standard Precipitation Index, SPI; and the Standard Precipitation Evaporation Index, SPEI. The indices were selected because of their direct link to floods and/or drought regimes. The evolution of the four climate indices between the reference period (1976–2005) and three future periods (2011–2040, 2041–2070, and 2071–2100) was assessed. The indices were calculated at 63 locations in the basin using the statistically downscaled output of eleven Regional Climate Models (RCMs) from the Coordinated Downscaling Experiment (CORDEX-AFRICA), under two Representative Concentration Pathways: RCP 8.5 (high emission scenario) and RCP 4.5 (moderate emission scenario). The indices were then interpolated over the basin using the Inverse Distance Weighting (IDW) method. Finally, the results are analyzed to provide an insight into the future of the basin in terms of flood and drought regimes, as well as related socioeconomic vulnerabilities in the basin.

## Materials and methods

### Available data

A total of 121 monthly measured precipitation times series and 17 measured minimum and maximum temperature time series were obtained from the Congo basin Water Resources Research Center (CRREBaC). The time series ranged from 1956 to 2019. The authors were able to secure the 1961–2019 daily rainfall observations at the Kinshasa-Mbinza station, but not at other locations in the basin. An alternative source of data was obtained from the WFDEI (Watch Forced Era Interim: Weedon et al. 2018) reanalysis data set; it contains daily time series of precipitation, minimum temperature, maximum temperature, relative humidity, wind speed, and solar radiation. While station data is generally considered more precise than the reanalysis data, there seemed to be many erroneous values in the observational data received by the authors. Therefore, the authors decided to discard any observed precipitation data where the annual totals were varied by more than 10% of the total calculated using WFDEI. The number of precipitation stations dropped to 50 after applying this screening method. The WFDEI data set was used at 12 stations located in areas without observational data to provide good spatial coverage within the watershed. Given that temperature estimates in the WFDEI database are more reliable than precipitation estimates, we decided to use the WFDEI time series at 130 locations.

Projected precipitation as well as minimum and maximum temperature from 11 climate change experiments obtained from the CORDEX (Coordinated Downscaling Experiment: Giorgi and Gutowski 2015) team (https://cordex.org/domains/region-5-africa/) were interpolated over 63 locations in the basin using the inverse distance weighting (IDW) method. Each time series spans from 1950 to 2100. Each climate change experiment consists of driving a regional climate model (RCM) by a global circulation model (GCM) under either Representative Concentration Pathway (RCP) 4.5 or RCP 8.5. RCPs are scenarios that include time series of emissions and concentrations of the full suite of greenhouse gases through to 2100. RCP 4.5 is a pathway (scenario) where radiative forcing peaks at approximately 4.5 W m^−2^ before 2100. RCP 8.5 is a higher intensity pathway for which radiative forcing reaches or exceeds 8.5 W m^−2^ by 2100. The characteristics of the climate change experiment (institution, driving GCM, RCM) are given in Table A2.1 (appendix 2). The spatial resolution of each RCM is approximately 50 km.

### Methodology

**Fig. 2:**
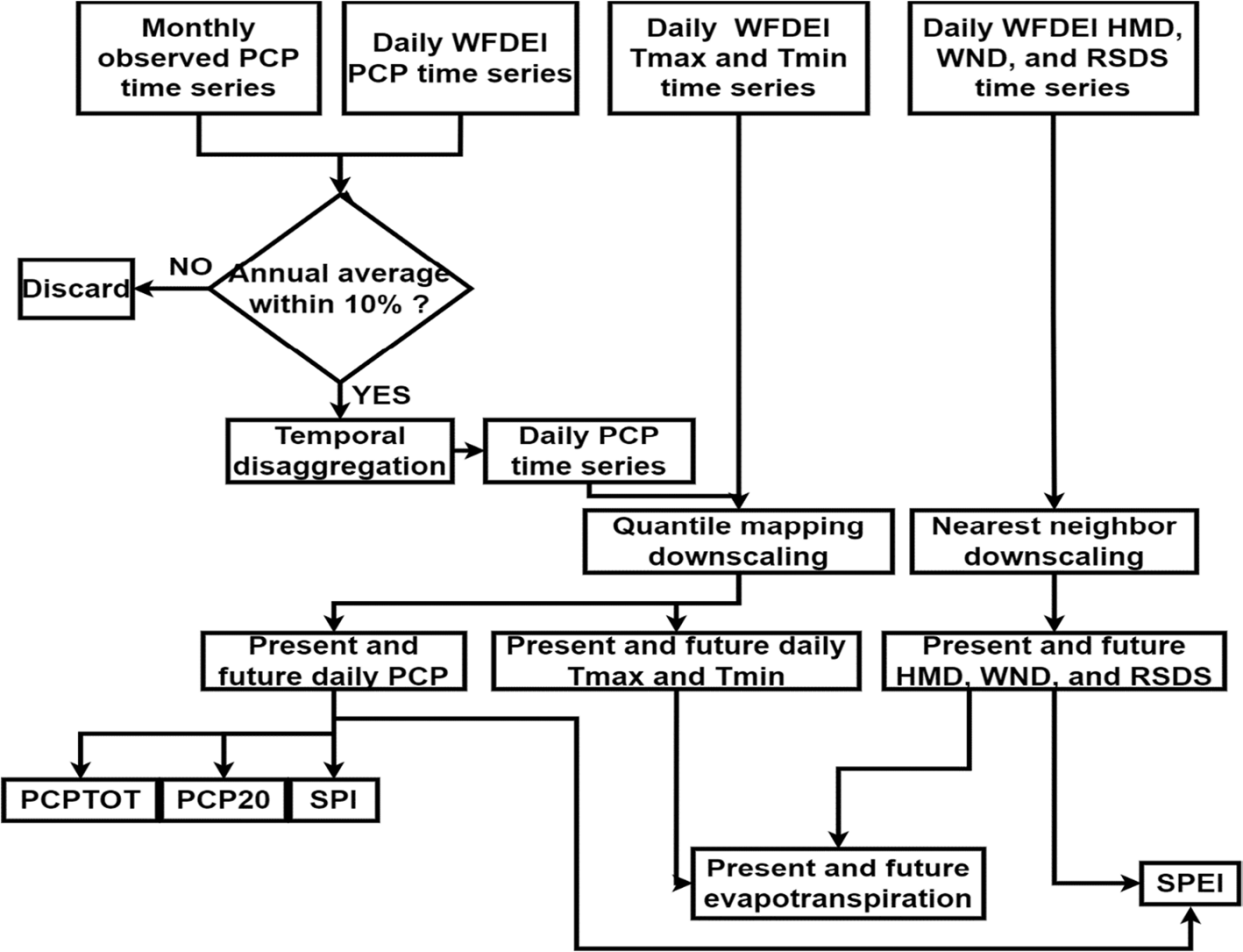
Methodology flowchart, where PCP stands for precipitation, HMD is humidity, WND is wind speed, RSDS is solar radiation, PCPTOT is total annual precipitation index, PCP20 is number of days where precipitation is above 20 mm index, SPI is the Standardized Precipitation Index, SPEI is Standardized Precipitation Evapotranspiration Index, and Tmax and Tmin are the maximum and minimum temperatures, respectively

The methodology is outlined in Fig. 2. In the first phase, available monthly observed precipitation and temperature time series are screened before the retained time series are temporally disaggregated using the fragment method to obtain daily time series. In the second phase, daily precipitation and temperature time series are statistically downscaled using quantile mapping and the outputs of eleven RCM/GCM combinations under both RCP 4.5 and RCP 8.5 scenarios. The result gives downscaled and bias-corrected precipitation and temperature time series representing past, present, and future conditions starting in 1976 and ending in 2100. Daily WFDEI humidity (HMD), wind speed (WND), and solar radiation (RSDS) time series are downscaled using the nearest-neighbor approach. The third and final phase involves developing four indices to evaluate the present and future daily precipitation (PCP) results from the downscaling. These indices are analyzed using a reference period and three future study periods to project the changes that will occur in the watershed in the near future. The reference period is between 1976 and 2005, future period 1 is between 2011 and 2040, future period 2 is between 2041 and 2070, and future period 3 is between 2071 and 2100. Present and future daily max temperature (TMP), daily min TMP, HMD, WND, and RSDS will be used to evaluate present and future evapotranspiration. Finally, HMD, WND, and RSDS combined with daily PCP will allow us to develop the SPEI index.

### Temporal disaggregation

Monthly observed rainfall time series were temporally disaggregated to the daily time step using the fragment method. The fragment method was first introduced by Harms and Campbell (1967) to increase the time scale of standardized historical rainfall. This conversion, which has no impact on monthly averages, was necessary as the calculation of PCP20 requires daily data. For each year of observed rainfall (Y_seasonal,obs_) from Equation [1], a year in WFDEI precipitation was chosen as follows: if the year was between 1979 and 2019, the same year was selected in the WFDEI data; if the year was anterior to 1979, the year in the WFDEI rainfall (Y_seasonal,WFDEI_) with the average rainfall value closest to that of the average observed rainfall was chosen. Daily precipitation of Y_sim_ at a given station STA_i_ was calculated using Equation [1] (Sittichok et al. 2016):

1 $${\mathrm{Y}}_{\mathrm{daily},\mathrm{sim},\mathrm{STAi}}= {\mathrm{Y}}_{\mathrm{daily},\mathrm{WFDEI},\mathrm{STAi}} ({\mathrm{Y}}_{\mathrm{annual},\mathrm{obs}}/{\mathrm{Y}}_{\mathrm{annual},\mathrm{WFDEI}})$$

This disaggregation method was used for all retained stations to generate daily PCP time series while maintaining the regional variations in rainfall across the watershed.

### Downscaling

The quantile mapping technique, also known as the quantile–quantile (Q-Q) transformation, was adopted to generate a climate variable’s statistical distribution to be as similar as possible to the statistical distribution of the corresponding observed variable during the historical period. According to Hakala et al. (2018), the quantile mapping technique outperforms other methods such as the delta-change approach, local intensity scaling, and power transformation. It has been proven to have higher success in reducing biases in GCM-RCM precipitation compared to other methods. First, the historical data set was separated into the calibration and validation data sets. The calibration period was composed of odd-number years in the reference period, while the validation period is composed of even-number years in the same period. Each month’s daily time series was extracted for the conforming periods from both the observational and RCM simulations (Angelina et al. 2015). Secondly, the empirical cumulative distribution functions of the observed calibrated period (F_OBS_) and the RCM outputs (F_RCM_) was developed. Finally, RCM simulations were corrected and generated for the validation period and for future periods using equation [2] for the transformation.

2 $${\mathrm{X}}_{\mathrm{CORR}}= {\mathrm{F}}_{\mathrm{OBS}}^{-1}({\mathrm{F}}_{\mathrm{RCM}}\left({\mathrm{X}}_{\mathrm{RCM}}\right))$$

X_CORR_ is the corrected variable and X_RCM_ is the extracted value from the raw RCM simulations. The probability mass function (pmf) of PCP occurrence and the probability distribution function (pdf) of PCP intensity were generated. If the pdf/pmf of the corrected value was more similar to that of the observations than that of the non-corrected value, then the Q-Q transformation was used on the future RCM simulations for the variable in question. Future values of HMD, WND, and SLR were downscaled using the nearest-neighbor approach. For each day in the future period (df), one day was chosen from the historical period from the same month (dh), and the absolute difference between the closest average temperature of df and dh was minimal. The HMD, SLR, and HMD of dh were then assigned to df.

### Climate indices

Four indices were employed to analyze the evolution of extreme events in the future: total annual precipitation (PCPTOT), number of days where precipitation is above 20 mm (PCP20), the Standard Precipitation Index (SPI) and the Standard Precipitation-Evapotranspiration Index (SPEI).

#### PCPTOT

The total annual precipitation index groups the annual total precipitation on wet days, where rainfall is above or equal to 1 mm of rain:

3 $${\mathrm{PCPTOT}}_{,j}= \sum_{\mathrm{i}=1}^{\mathrm{I}}{\mathrm{R}.\mathrm{R}.}_{\mathrm{ij}}$$

where RRij represents the daily precipitation on wet days, for which i represents the number of days in a certain time period j:

#### PCP20

This index is the average number of days per year when the rainfall is above 20 mm. The index of PCP20 is a standardized climate extreme index (https://www.climdex.org/learn/indices/#index-R20mm). And it is also included in the definitions of the 27 core indices (http://etccdi.pacificclimate.org/list_27_indices.shtml)

4 $${{PCP20}_{,j}}_{\mathrm{j}} = \sum_{\mathrm{i}=1}^{\mathrm{I}}{(\mathrm{RR}}_{\mathrm{ij}}>20\mathrm{ mm})$$

#### SPI

SPI is a widely used index used to characterize meteorological drought at various timescales. It was first proposed by Mckee et al. (1993) and has been used by many authors in various regions around the World (e.g., Bodian 2014). It is, in essence, a standardizing transform of the probability of observed precipitation (Guttman 1998):

5 $$SPI=\frac{\mathrm{PCPTOT},\mathrm{i}-\mathrm{PCPTOT},\mathrm{m}}{\mathrm{Si}}$$

where PCPTOT,i represents the sum of precipitation for a given year i; PCPTOT,m represents the mean value of precipitation over the reference period; and Si is the standard deviation of precipitation aggregated at the user-defined time scale. In this paper, SPI is calculated on an annual time scale. It can be deduced from Eq. 5 that the average SPI over the reference period is equal to zero.

Degrees of humidity and drought can be assessed using the SPI classes outlined in Table 1.

**Table 1:** Classes of SPI

SPI class	Degree of humidity or drought
SPI > 2.0	Extreme humidity
1.5 < SPI < 2.0	High humidity
1.0 < SPI < 1.5	Moderate humidity
-1.5 < SPI < 1.0	Moderate drought
-2.0 < SPI < -1.5	Severe drought
SPI < -2.0	Extreme drought

#### SPEI

SPEI is an extended version of SPI. SPEI is calculated using the same method as for the SPI, which defines the degree of drought in different classes (Lebel and Ali 2009). The fundamental difference between these two indicators is that SPI is calculated based on precipitation, while SPEI is based on the difference between precipitation and potential evapotranspiration (PCP-ET_0_). Then, this cumulative value of PCP- ET_0_ over m months, the log-logistic law is adjusted to three parameters (Vicente-Serrano et al. 2010). SPEI for a given period can be calculated from:

6 $$SPEI={CDF}_{norm(\mathrm{0,1})}^{-1}\left({CDF}_{log-logistic}\left(PCP-{ET}_{0}\right)\right)$$

where ${CDF}_{norm(\mathrm{0,1})}^{-1}$ is the inverse cumulative distribution function of the normal distribution with mean zero and standard deviation 1 and ${CDF}_{log-logistic}$ is the cumulative distribution function of the log-logistic distribution fitted to PCP- ET_0._ The reference evapotranspiration (ET_0_) value used in this paper is the reference evapotranspiration proposed by Neitsch et al. (2011) for alfalfa. The method requires various inputs such as solar radiation, air temperature, relative humidity, and wind speed. The equations needed to calculate the reference evapotranspiration are given in the supplementary materials (Appendix 1). The degree of humidity and drought can be assessed using SPEI with the same classes as for SPI presented in Table 1.

### Statistical testing

The two-sample *t* test (Snedecor and Cochran 1989) was used to assess the significance of changes in PCPTOT and PCP20 between the reference period and future periods. The purpose of the *t*-test is to compare the means of two independent data sets. It takes a sample from each of the two sets and creates the problem statement by assuming a null hypothesis that the two means are equal. Based on the applicable formulas, certain values are calculated and compared to the standard values and the assumed null hypothesis is accepted or rejected accordingly. Given that multiple time series are available for each climate index and that each series’ mean and standard deviation are different, each time series was standardized using its mean and standard deviation of the reference period. The standardized series were then combined prior to performing the *t* test.

## Results and discussion

A total of 63 stations were retained for precipitation (51 daily time series were obtained by disaggregation and 12 were added from the WFDEI precipitation), and 121 time series of maximum and minimum temperature from the WFDEI reanalysis were downscaled using the quantile mapping downscaling method. Humidity, solar radiation, and wind speed were downscaled using the nearest-neighbor technique. The four indices were calculated for the reference period 1, period 2, and period 3. The results were interpolated over the watershed using the inverse distance method. The results of the analysis are discussed below.

### Impact of the temporal disaggregation on the magnitudes and trends of PCP20, PCPTOT, SPI, and SPEI

The impacts of the temporal disaggregation were assessed on the climatic indices at the only station at which the authors were able to secure daily observed data: the Kinshasa-MBINZA station (latitude =  − 4.36; longitude = 15.25). The results of the assessment are shown in Appendix A3. The difference in average annual precipitation between WFDEI and the observations is only 28 mm (2%), and the disaggregated time series have the same mean as the observation. It was found that the number of wet days is underestimated in both WFDEI and the disaggregated time series. This led to a significant underestimation of PCP20 when the disaggregated time series were used. In the 1979–2013 period where there are valid values in all three data sets, PCP20 was 25.72 days per year in the observations, 5.85 days per year in the WFDEI data set, and 6.5 in the disaggregated data set. For the Kinshasa-Mbinza station, the magnitude of PCP20 will be severely underestimated when the disaggregated time series is used. However, the temporal disaggregation did not seem to affect the trend of that index. The magnitudes and trends of other indices (PCPTOT, SPI, SPEI) were only marginally affected.

### Spatial distribution of indices in the reference period

**Fig. 3:**
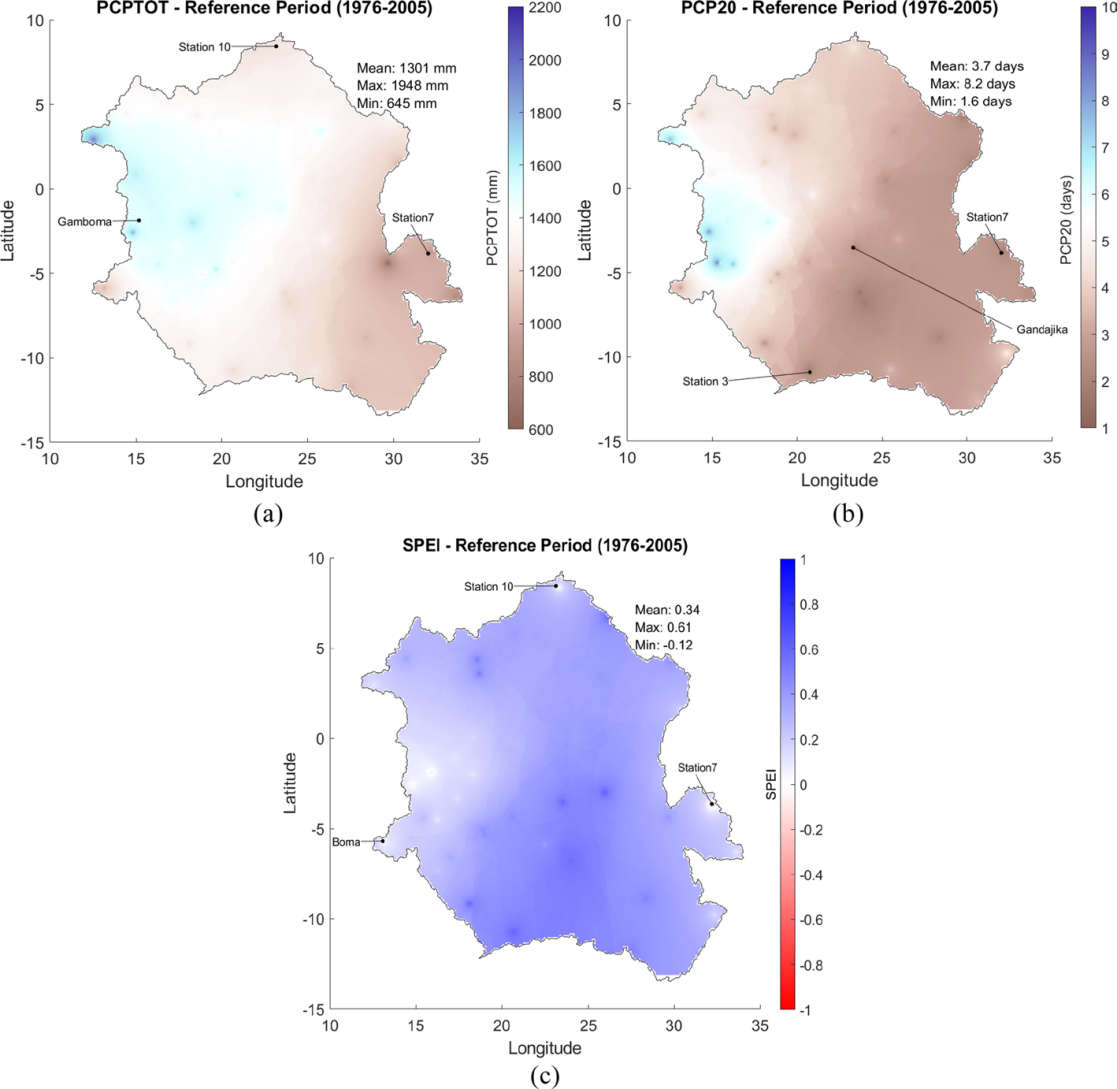
Spatial distribution of multimodel average (**a**) PCPTOT (mm), (**b**) PCP20 (days), and (**c**) SPEI in the Congo basin for the reference period of 1976–2005

Figure 3(a) shows the spatial distribution of the average annual total precipitation in the entire watershed for the reference period (1976–2005). The average annual precipitation ranges from 645 to 1000 mm/year in the south-eastern part of the basin up to 1948 mm/year. The highest average annual precipitation values are observed in the center and western parts of the basin. Figure 3(b) shows the spatial distribution of the average PCP20 (a proxy for the frequency of high-intensity precipitation) for the reference period, where the average annual number of days with rainfall above 20 mm ranges between 1.6 days and 8.2 days. The highest rainfall intensity occurs close to the western border of the Congo basin. Figure 3(c) depicts the spatial distribution of SPEI in the Congo basin for the reference period, where values range from − 0.12 to 0.61. SPEI in the reference period suggests a dryer climate in the central west compared to the rest of the watershed. SPI in the reference period is by definition zero throughout the entire basin.

### Projections for PCPTOT

**Fig. 4:**
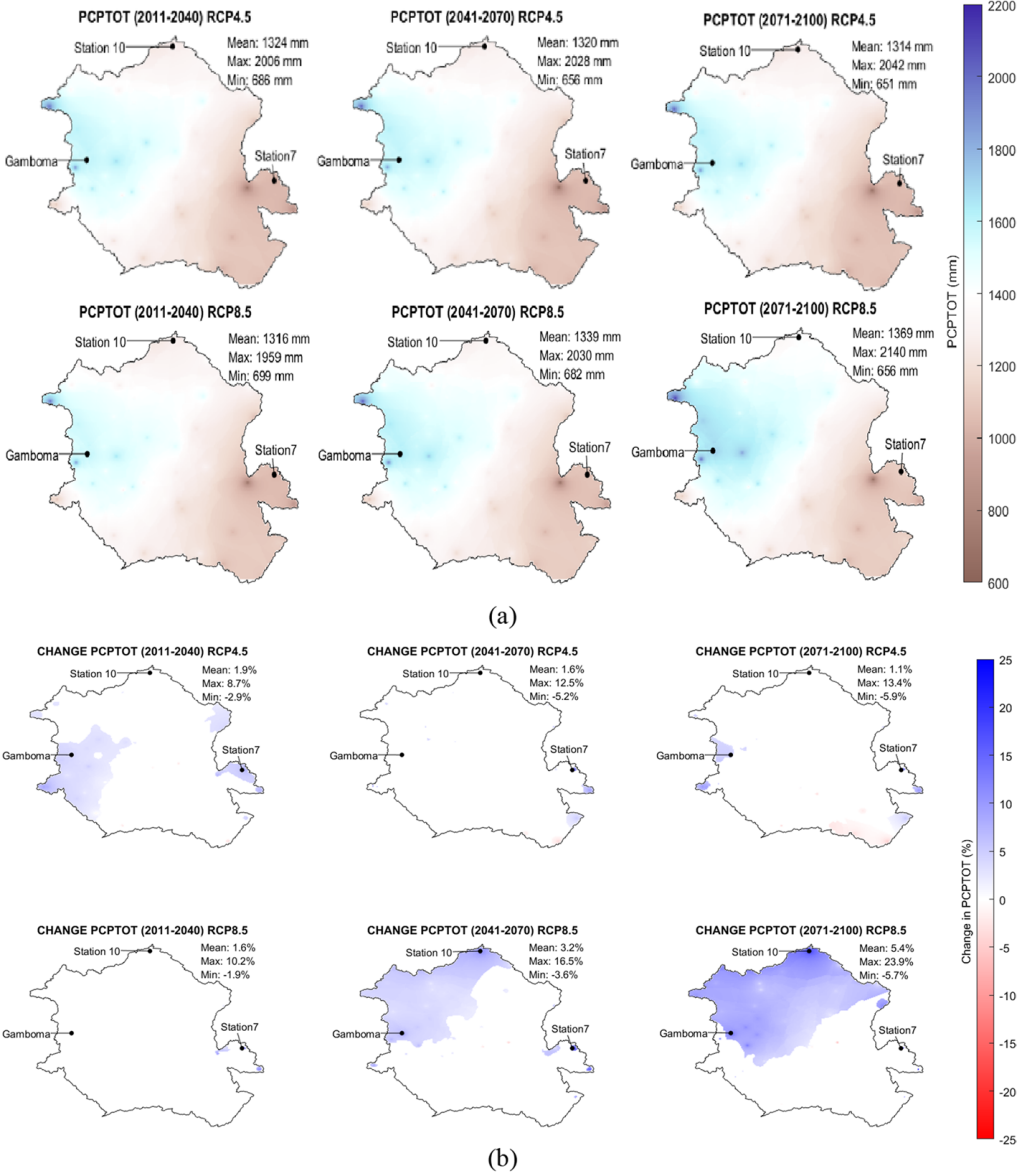
Spatial variation of multimodel averages of (**a**) PCPTOT and (**b**) the change of PCPTOT for three future periods ((2011–2040), (2041–2070), (2071–2100)) under RCP 4.5 and RCP 8.5; white spaces correspond to non-significant changes

Figure 4(a) shows the spatial distribution of the multimodel average total precipitation and (b) the percentage change in the total precipitation that can be expected in the basin for the three study periods under RCP 4.5 and RCP 8.5 scenarios. Under RCP 4.5, the spatial average of the percent change in PCPTOT is very small: it is equal to 1.9% in period 1, 1.6% in period 2, and 1.1% in period 3. Under RCP 8.5, it increases from 1.6% in period 1 to 3.2% in period 2. It reaches 5.4% in period 3. The highest increases are expected in the northern part of the basin in period 3 under RCP 8.5

While the spatial average of the percent change in PCPTOT is not high (as shown in Fig. 4(b)), local changes can differ significantly from the average. Figure 4(b) shows that changes in PCPTOT are generally non-significant under RCP 4.5, except from the extreme western and eastern regions of the basin where slight localized increases are expected in periods 1, 2, and 3. An increase in PCPTOT is expected under RCP 8.5 in the north-western and northern regions of the basin in periods 2 and 3, respectively.

Under RCP 4.5, the increase in the spatial average of PCPTOT is modest (under 2%) and slightly decreasing from period 1 to period 3, but the maximum and minimum changes in the basin become more extreme with time (the minimum value gradually decreases from − 2.9% in period 1 to − 5.2% in period 2 and − 5.9% in period 3; the maximum value gradually increases from 8.6% in period 1 to 12.5% in period 2 and to 13.4% in period 3).

Under RCP8.5, the average PCPTOT displays an increasing trend with time. Similarly as under scenario RCP4.5, the maximum value of PCPTOT displays an increasing trend (10.2% in period 1, 16.5% in period 2 and 23.9% in period 3) while the minimum value of PCPTOT displays a decreasing trend (− 1.9% in period 1, − 3.6% in period 2, and − 5.7% in period 3). Changes in PCPTOT are generally not significant in period 1, while significant increases are observed in the North-west part of the basin (period 2) and in the northern half of the basin (period 3).

**Fig. 5:**
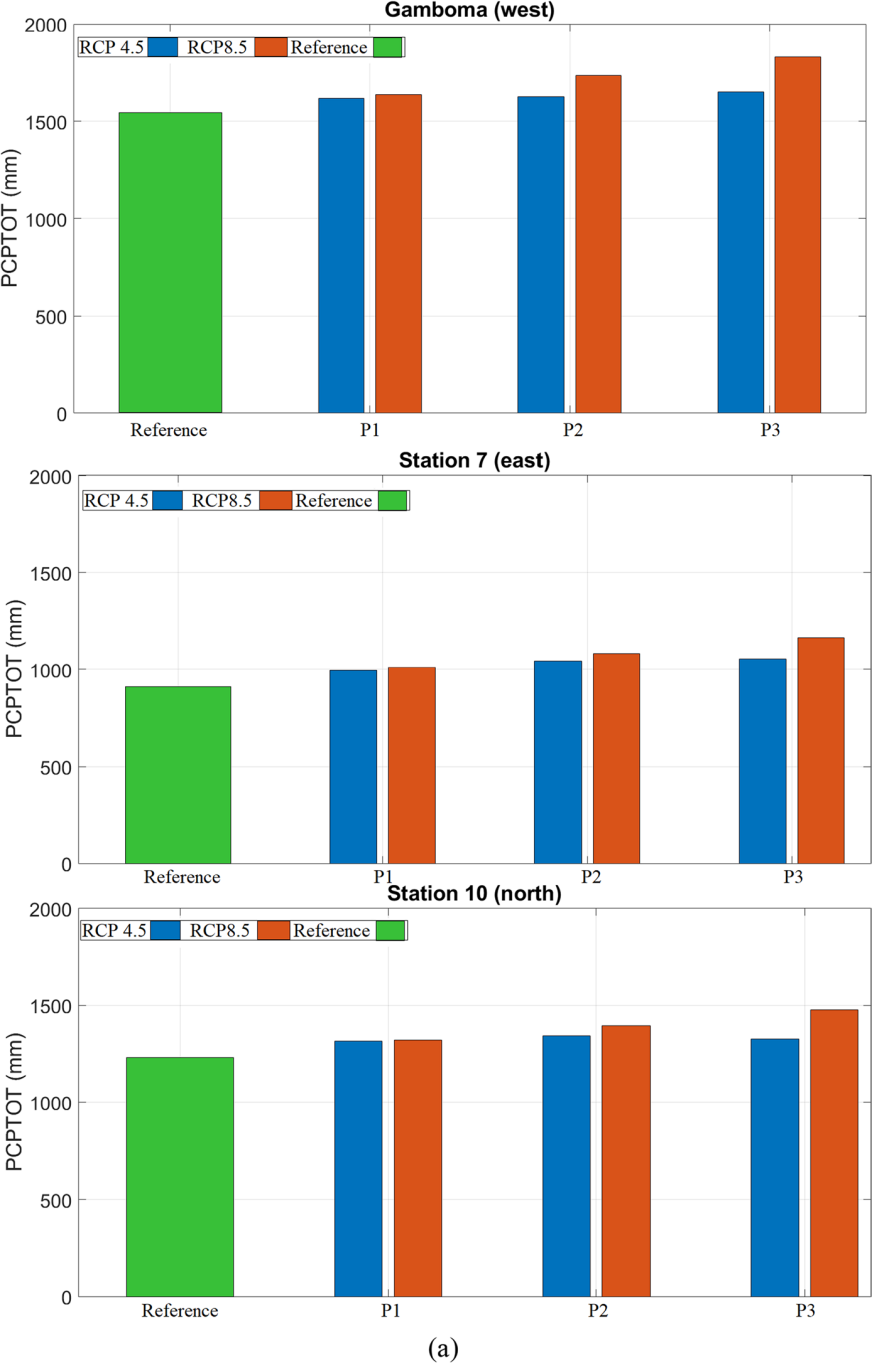

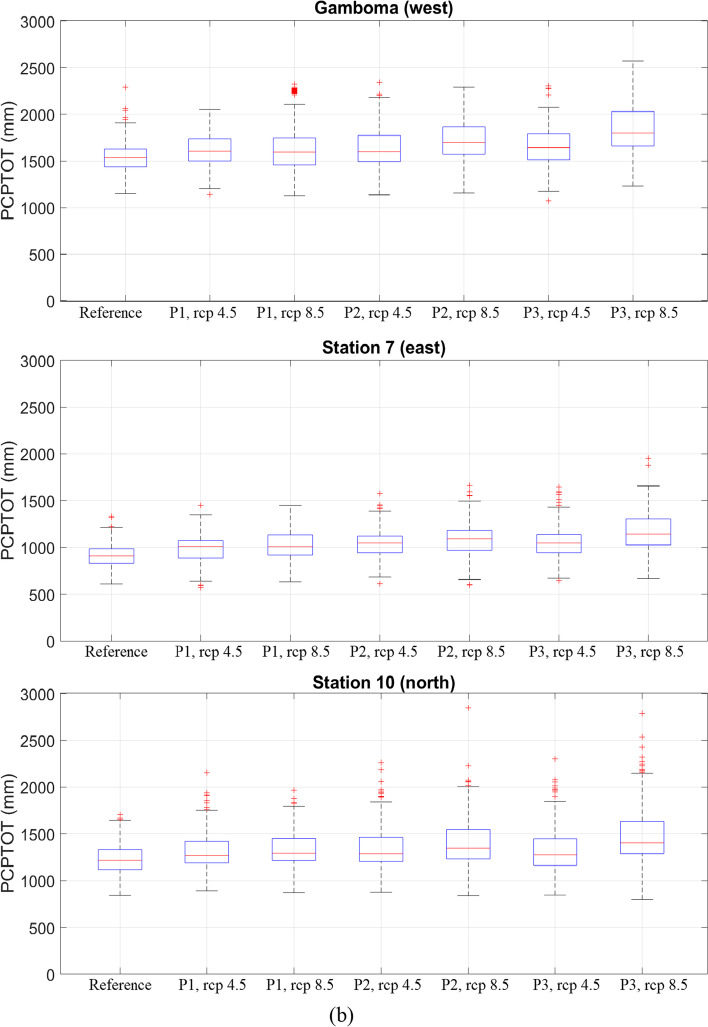
(**a**) Average values and (**b**) boxplots of *PCPTOT* under RCP 4.5 and RCP 8.5 scenarios for the reference period (Reference: 1976–2005) and three future periods (P1: 2011–2040), (P2: 2041–2070), (P3: 2071–2100) for selected locations in the Congo basin: station 7 (east), station 10 (north), and Gamboma (west).

Among the 63 stations used in this study, station 7 (east), station 10 (north), and the town of Gamboma (west) experienced the greatest increase in PCPTOT and were selected for further analysis. Figure 5 shows boxplots of the average annual total precipitation, PCPTOT at these three stations. PCPTOT increases at all three stations independently of the RCP scenario.

### Projections for PCP20

**Fig. 6:**
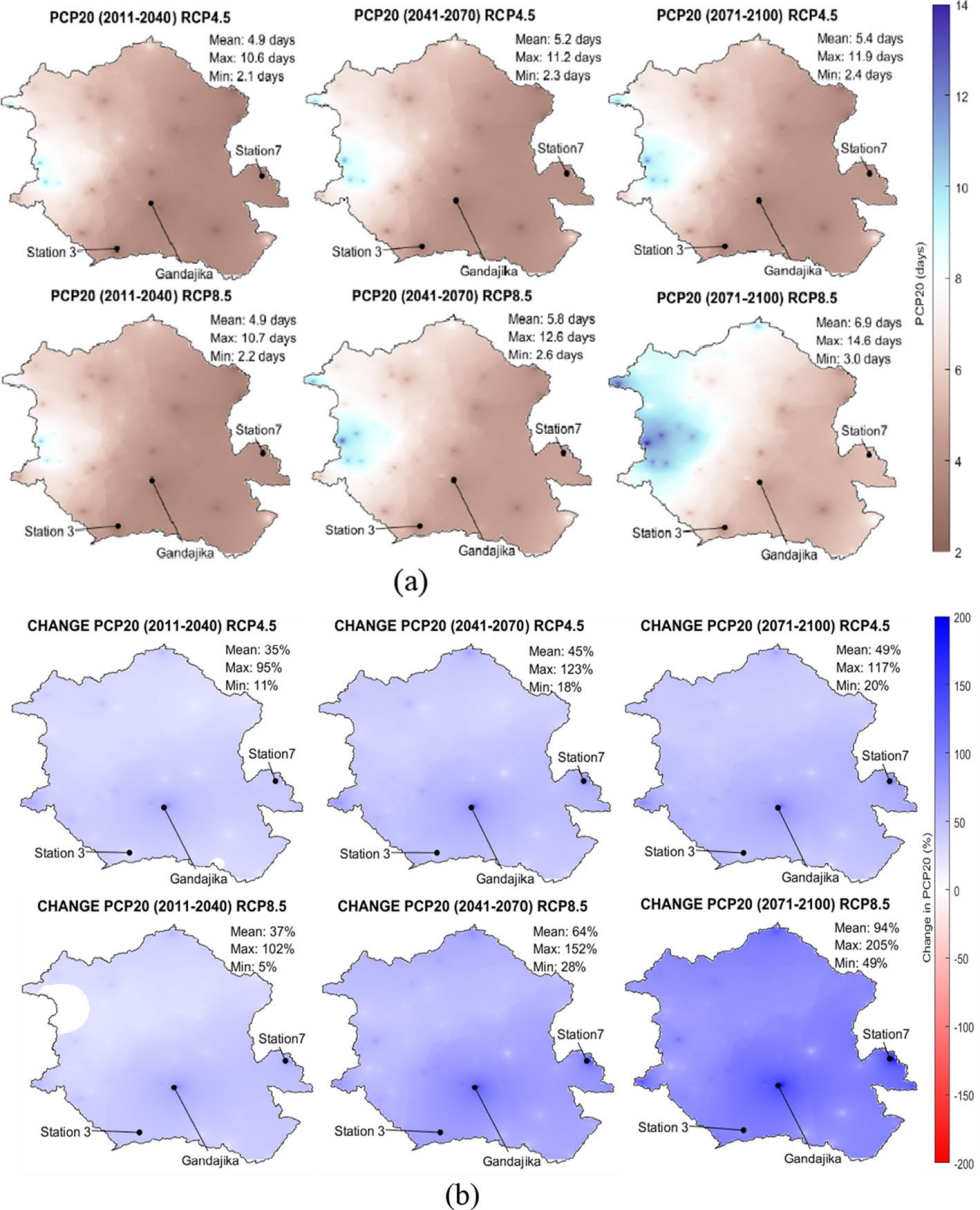
Spatial variation of (**a**) average PCP20 and (**b**) average change of PCP20 for three future periods ((2011–2040), (2041–2070), and (2071–2100)) under RCP 4.5 and RCP 8.5

Figure 6(a) shows the spatial distribution of the multimodel average PCP20, while Fig. 6(b) shows the percent change of PCP20 under RCP 4.5 and RCP 8.5 scenarios for the three future study periods. The magnitude of PCP20 in these figures should be considered cautiously since the authors have found that at one particular station (Kinshasa-MBinza), and PCP20 is underestimated when temporally disaggregated time series are used (see appendix A3).

Under RCP 4.5, there is a substantial increase in the average PCP20: 35% in period 1, 45% in period 2, and 49% in period 3. Compared to all other stations used in the study, the increase of PCP20 is particularly important at three locations: the city of Gandajika, station 3, and station 7 (Fig. 6). In period 3, this increase intensifies and spreads out slightly in the noted areas. Under RCP 8.5, there is a drastic increase in the areal average PCP20 (37% in period 1, 64% in period 2 and 94% in period 3). For period 3, most of the basin experiences intense rainfall events, as PCP20 increases by 49–205%. This rise in the frequency of high-intensity rainfall events could likely lead to increased flooding, which could have various impacts on the watershed's hydrological regime. This includes negative impacts on socioeconomics, agriculture, biodiversity, hydropower, and the environment.

**Fig. 7:**
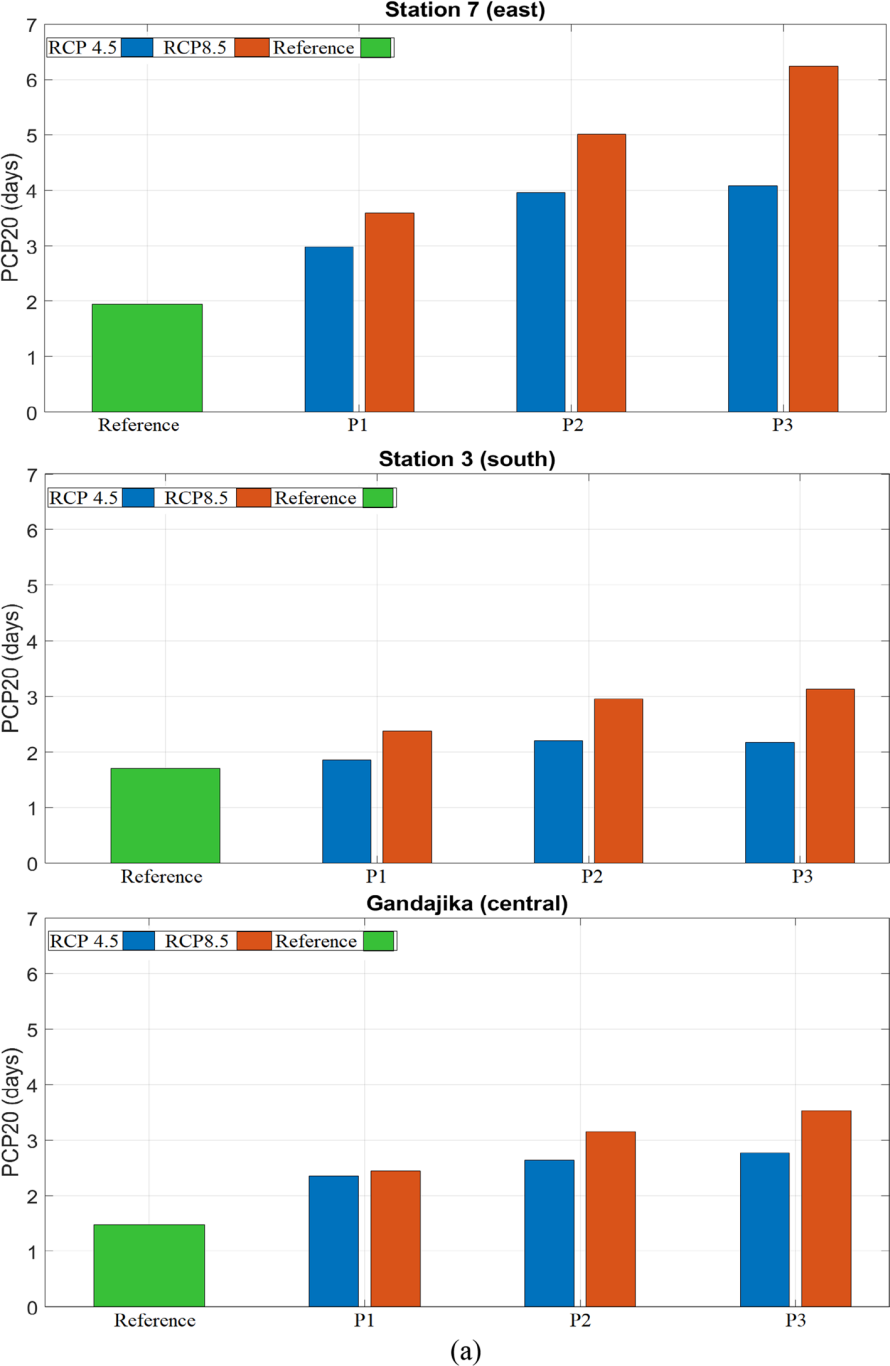

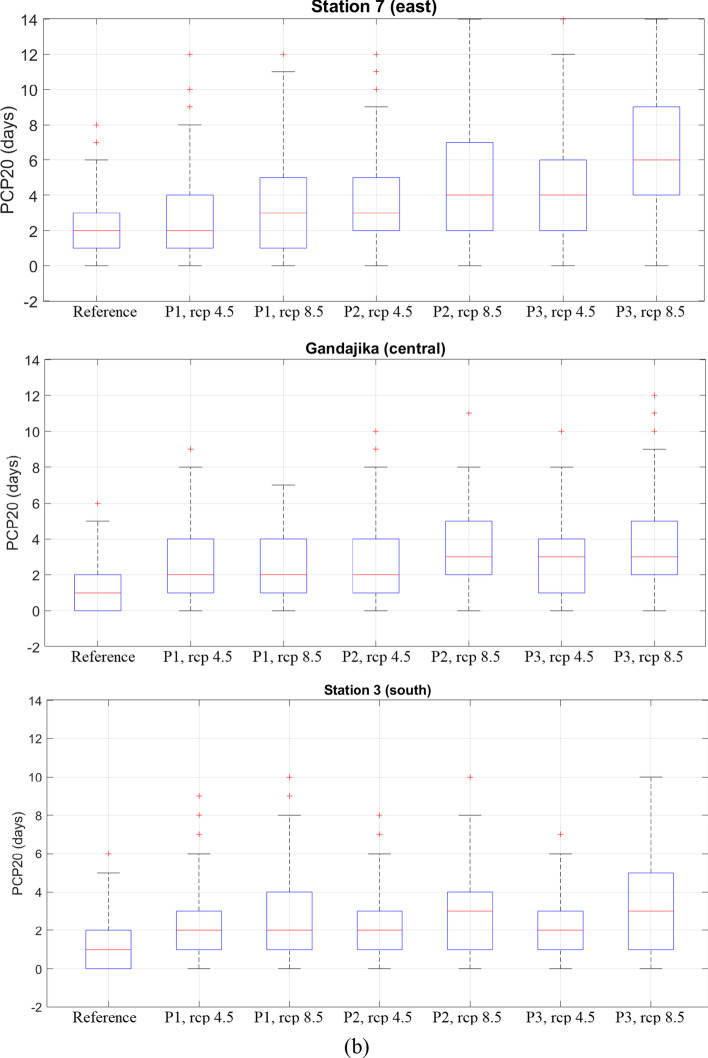
(**a**) Average values and (**b**) boxplots of *PCP20* under RCP 4.5 and RCP 8.5 scenarios for the reference period (1976–2005) and three future periods ((2011–2040), (2041–2070), (2071–2100)) for selected locations in the Congo basin: station 7 (east), Gandajika (central region), and station 3 (south).

Stations in the watershed that have the highest increase in PCP20 and show potential for future floods or environmental perturbations include station 7 (east), station 3 (south), and the town of Gandajika (central region). The boxplots and charts in Fig. 7 show changes in PCP20 for both RCP scenarios at the locations of interest. Out of the three stations, station 7 will likely undergo the most severe increase in heavy rainfall events in the entire basin, with an increase of approximately 2.5–4.5 days of intense rain per year, according to RCP scenarios 4.5 and 8.5, respectively. These changes should however be interpreted with care as PCP20 in the disaggregated time series is underestimated according to the assessment in Appendix A3.

### Projections for SPI

**Fig. 8:**
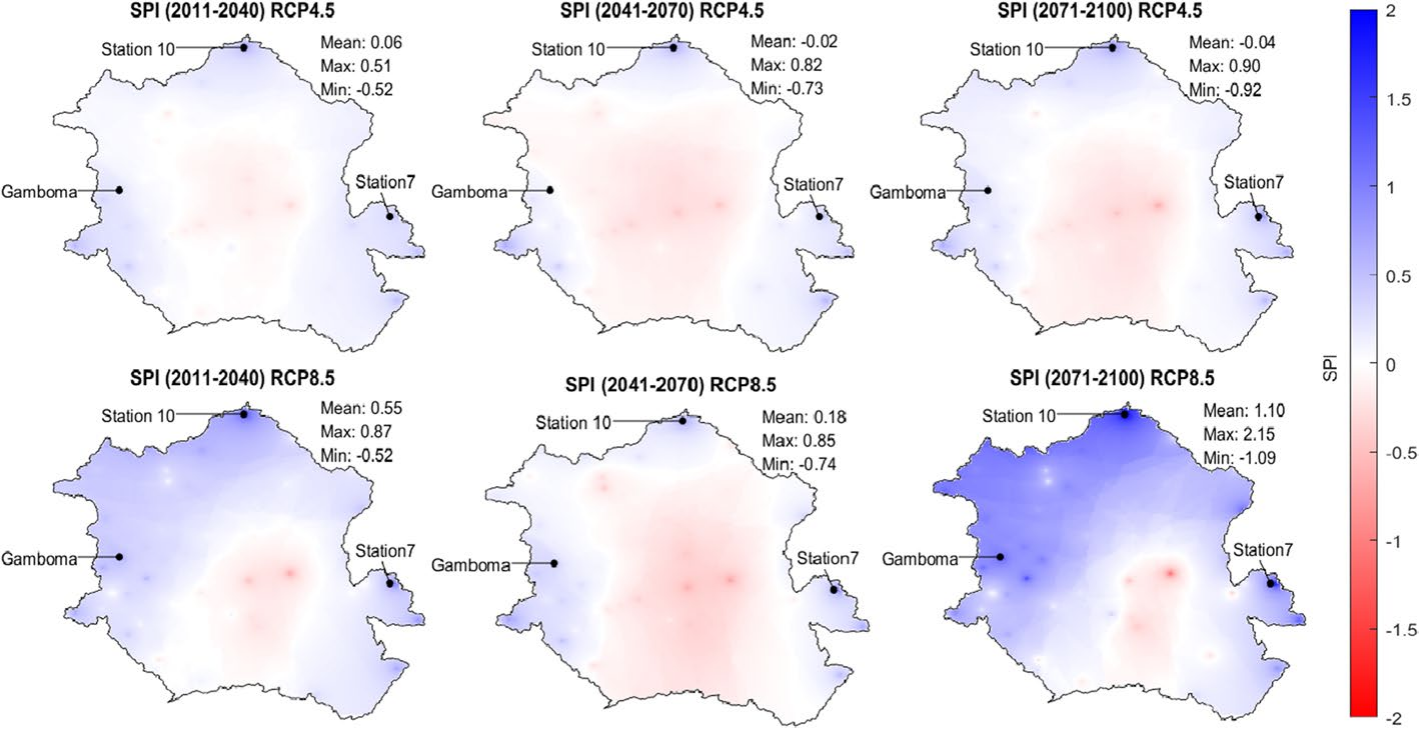
Spatial variation of multimodel average SPI for three future periods ((2011–2040), (2041–2070), (2071–2100)) under RCP 4.5 and RCP 8.5

The spatial distribution of the multimodel average SPI for the three future periods and the two emission scenarios is presented in Fig. 8. As expected, it closely follows that of annual precipitation. There is a progressive increase of SPI in the north, east, and western extremities of the Basin and a decrease of SPI in the center of the Basin for both scenarios. The most extreme changes occur under RCP 8.5 in period 3. These results suggest that the center of the Basin will become dryer, while the north, east, and western extremities of the Basin will become wetter in the future.

Under RCP 4.5, the spatial average of SPI goes from 0.06 in the period 1 to − 0.02 in period 2 and − 0.04 in period 3. Under RCP 8.5, it decreases from 0.55 in period 1 to 0.18 in period 2 and increases again to 1.1 in period 3. This is consistent with the findings that total precipitation changes are expected to be marginal.

### *Projections for SPEI*

**Fig. 9:**
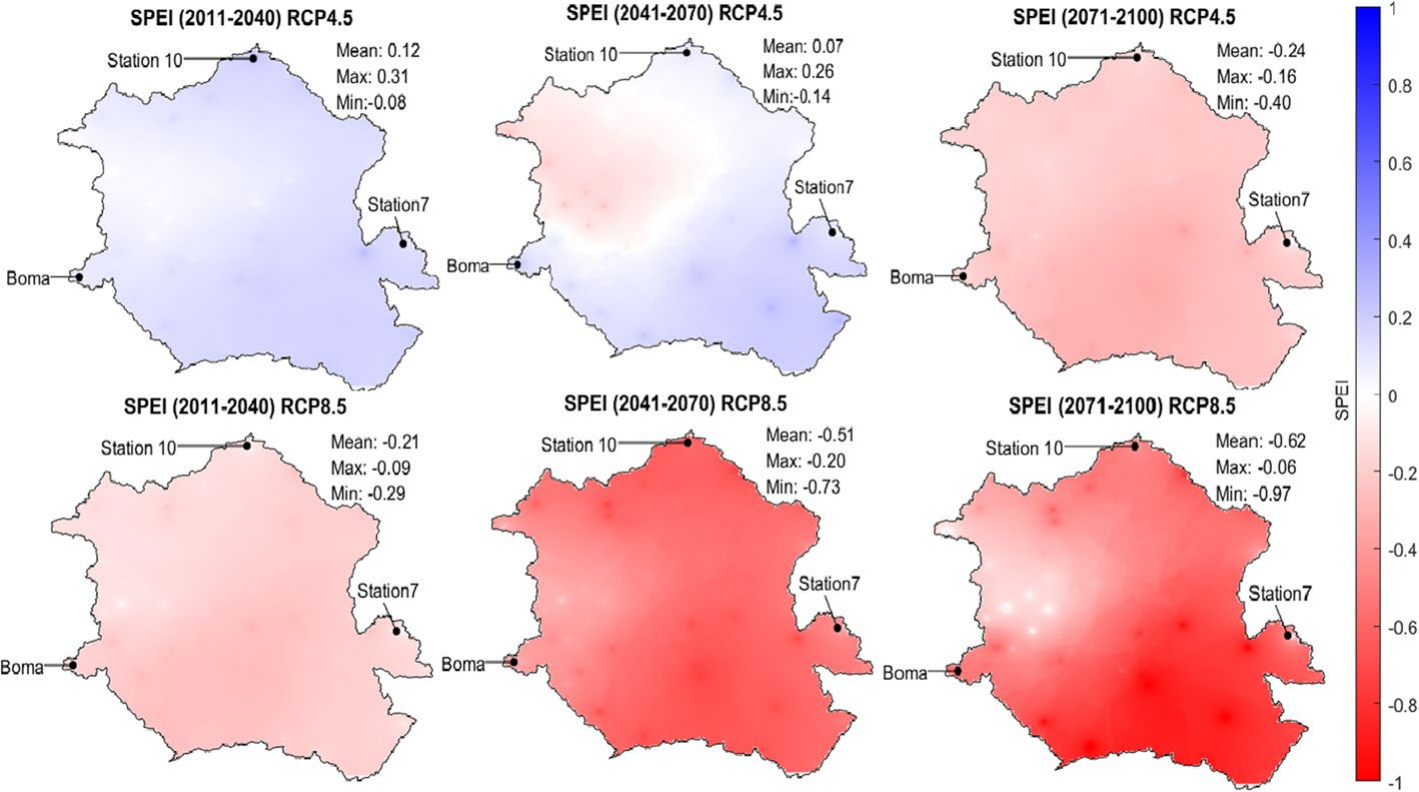
Spatial variation of multimodel average SPEI for three future periods ((2011–2040), (2041–2070), and (2071–2100)) under RCP 4.5 and RCP 8.5

The SPEI index accounts for precipitation and potential evapotranspiration (PET) and is used mostly to determine drought and dry conditions. Figure 9 shows changes in the average multimodel SPEI which are depicted on spatial distribution maps of the Congo basin for the three future periods under the two RCP scenarios. Under RCP 4.5, the spatial average of SPEI decreases from 0.12 in period 1 to 0.07 in period 2 and − 0.24 in period 3, on average. Under RCP 8.5, the spatial average decreases from − 0.21 in period 1 to − 0.51 in period 2 and − 0.62 in period 3. Values of SPI range from − 1.09 to 2.15, while SPEI values range from − 0.06 to − 0.97, respectively, for period 3, under RCP 8.5. This difference is due to the consideration of evapotranspiration. The fundamental difference between these two indicators is that SPI is calculated based on precipitation, while SPEI is based on the difference between precipitation and potential evapotranspiration. Although precipitation was shown to increase over most of the basin, SPEI systematically decreases independent of the emission scenario. That means that the increase in evapotranspiration (due to a rise in temperature caused by climate change) will offset the increase in precipitation in areas where rainfall amounts are expected to increase. It will exacerbate the severity of droughts in areas where precipitation is expected to decrease. As expected, the decrease in SPEI is less severe under RCP 4.5 than under RCP 8.5. Finally, locations that are most impacted by extreme changes/evaporation increases include station 10 (north) and various stations in the southeast regions of the basin.

**Fig. 10:**
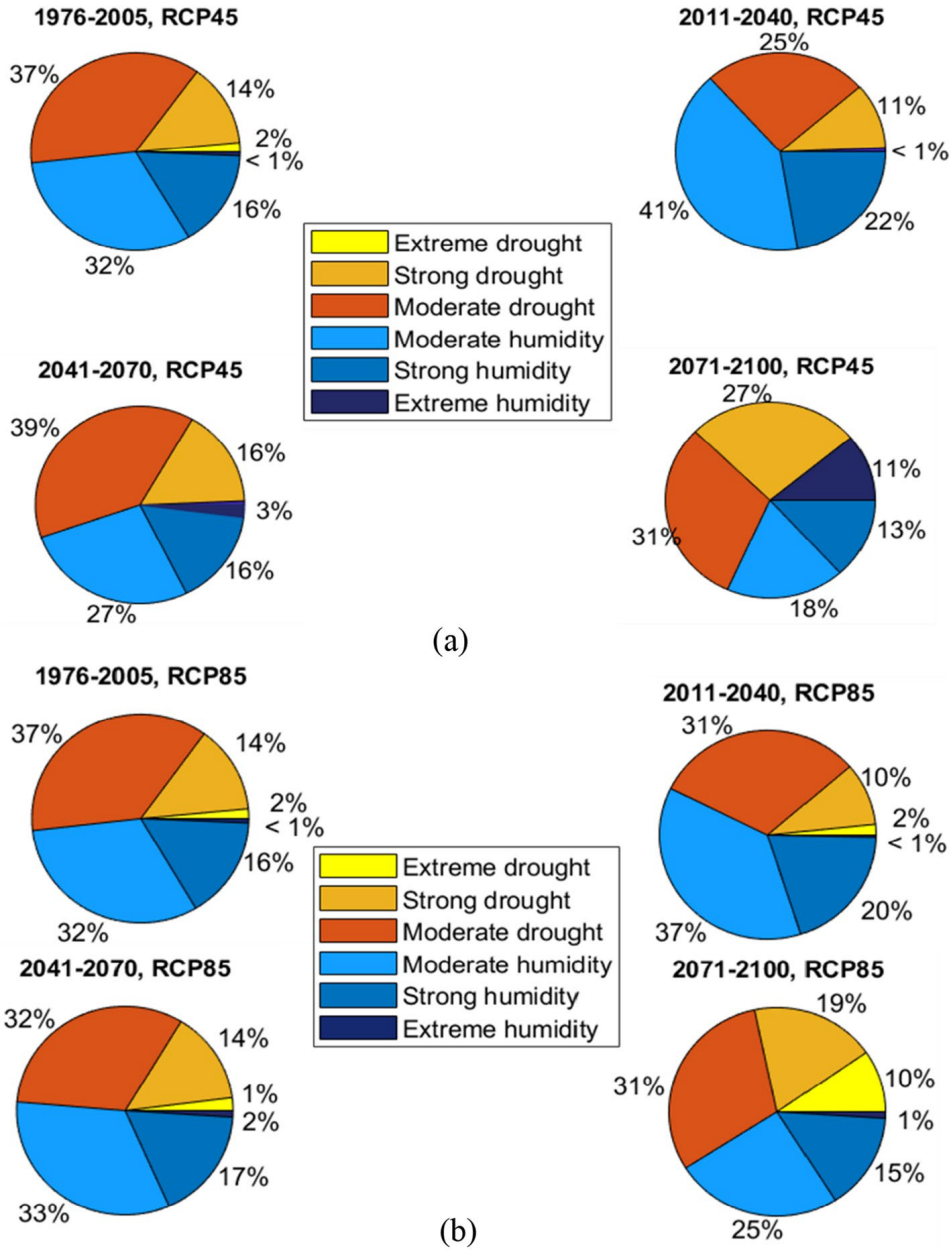
Multimodel average SPEI pie chart at Station 10 under RCP 4.5 (**a**) and RCP 8.5 (**b**) for the reference period and three future periods ((2011–2040), (2041–2070), and (2071–2100))

The next part of the results focuses on areas that experience the most significant changes in SPEI. These areas include station 10 (north), represented in Fig. 10. This figure shows the relative frequency of SPEI classes for the reference period and the three future periods under (**a**) RCP 4.5 and (**b**) RCP 8.5.

For station 10, humidity decreases throughout all the periods, whereas drought frequency increases significantly. The frequency of extreme drought at station 10 increases from 2 to 10% under RCP 8.5. Simultaneously, the frequency of extreme humidity increases from less than 1% to 11% under RCP 4.5.

### Discussion

The climate regime in the Congo River basin directly affects the livelihood of 75 million people living in the watershed and the health of one of the planet’s largest rainforests and carbon sink with direct links to the global climate. However, the basin is understudied, partly because of the lack of observational data (Creese et al. 2019). While annual and seasonal precipitation and evaporation have received some attention in the literature, the authors are not aware of any projection of extreme daily precipitation, probably because reliable daily precipitation records are rare. While most studies suggest a stagnation or an increase in total rainfall in the future, little information is available about whether that increase would offset the increase in evaporation resulting from higher air temperatures. The lack of reliable daily precipitation records was tackled in our study by disaggregating monthly observed precipitation time series using the fragment method.

Multiple studies (Wong et al. 2017; Beck et al. 2017; Berg et al. 2018) have successfully used WFDEI, which performs well when used to drive hydrological models. However, in a study over West Africa, Satgé et al. (2020) found that reanalysis data sets such as WFDEI present the lower statistical scores at the daily time step compared to satellite-based precipitation datasets but their performance increases at the monthly time step. The impact of the temporal disaggregation on the magnitude and trends of PCP20, PCPTOT, SPI, and SPEI was examined at the Kinshasa-Mbinza station, where daily precipitation was available. For that particular climate station, the temporal disaggregation led to an underestimation of PCP20, but did not affect the trend of PCP20 in the future. Unfortunately, the conclusion cannot be generalized to other locations in the Congo basin. Therefore, the values of PCP20 presented this paper and in Figs. 6 and 7 are most likely underestimated and hence should be interpreted with caution.

Despite the limitations in the datasets used, the results in this paper strongly suggest that there will be a moderate increase in total precipitation in most of the basin (especially in the northern half of the basin from the mid-century under RCP 8.5). The change in total precipitation in the central and southern parts of the basin was not found to be significant, except for a small area in the South-east where a small decrease (up to -6%) is expected under RCP4.5 in period 3. These findings are consistent with those of Tshimanga and Hughes (2014), Haensler et al. (2013), and Mba et al. (2018), who found that annual total precipitation amounts over the Congo basin are not likely to change severely in the future. Creese et al. (2019) found a moderate trend towards wetting over the Congo basin in the CMIP5 models. The results of this study also suggest that an increase in the number of days with high-intensity rainfall (PCP20) is expected over the entire watershed, suggesting a possible increase in flash flood events. According to our PCP20 projections, all locations of the Congo basin will undergo a higher frequency of heavy rainfall. This means that local people will likely observe more intense and frequent periods of flooding in the foreseeable future where preparedness for such events through systematic policy implementation for -disaster risk reduction is essential. At the same time, as our projections for SPEI show, the frequency of droughts will increase even at locations where total precipitation is expected to increase in the future. This means that increased evaporation resulting from higher temperatures will offset the rise in total precipitation at these locations. The result will be an exacerbation of extreme lows and extreme highs, leading to impacts on both ends of the spectrum. The fact that flash floods and drought are expected to occur in the same areas is not a contradiction as the two events have different time scales (a few hours to a few days for floods, a season to several years for a drought). Therefore, economic losses may increase unless concrete preventative measures are put into place to minimize flooding risks. Finally, the evolution of SPEI over time shows that increased evaporation resulting from rising temperatures will outweigh the overall increase in PCP. As a result, local people in the basin will face an increase in the annual drought frequency, and consequently, this could lead to a possible collapse in food security in the region.

The projected increase in drought frequency may have impacts beyond the limits of the basin by modifying the spatial distribution of forested areas, the biomass production in these areas, and hence their carbon sequestration capabilities. Hubau et al. (2020) listed droughts as a potential reason for an observed carbon sink saturation that started after 2010 in African tropical forests. The loss of tropical forest carbon sequestration policy would have far-reaching consequences on the global climate and climate change adaptation/mitigation policies. It is therefore of crucial importance to further look into the biophysical impact of the projected change of climate regime on a range of sectors: forest distribution and ecosystem services, including carbon sequestration; hydrological regimes; agriculture and cattle grazing, etc.

## Conclusions

Four climate indices (total annual precipitation, PCPTOT; number of days where PCP is above 20 mm, PCP20; the Standardized Precipitation Index, SPI; and the Standardized Precipitation-Evaporation Index, SPEI) were selected and estimated using the downscaled outputs from eleven regional climate models under representative concentration pathways RCP 4.5 (moderate greenhouse gas emissions scenario) and RCP 8.5 (high greenhouse gas emissions scenario). Relative changes in these indices were analyzed in three future periods (2011–2040, 2041–2070, and 2071–2100) and compared to the historical reference period (1976–2005). It was found that (a) under RCP 8.5, there is an expected increase in total PCP and SPI, in the north-western and northern region of the basin in periods 2 and 3, respectively; (b) the maximum increase in PCPTOT (+ 24%) and the minimum value of SPEI (− 0.97) were both projected under RCP 8.5 in the period from 2071 to 2100; and (c) PCP20 will increase independently of the period and scenario, in the entire watershed. Under RCP 8.5 in the 2071–2100 period, PCP20 will increase by 94% on average over the whole watershed. An increase in the number of days with high-intensity rainfall is expected over the entire watershed, suggesting a possible increase in flood events. Finally, the SPEI index points to an increase in drought throughout most of the basin, as the increase in evaporation resulting from rising temperatures will outweigh the overall rise in PCP. This study portrays a basin that will undergo an intensification in drought and flood frequency in the future. It can also be used to guide the development of adaptation approaches towards enhancing flood and disaster management, vulnerability mapping, flood monitoring systems, early warning systems, development of infrastructure, and other socioeconomical aspects.

## Supplementary Information

Below is the link to the electronic supplementary material.

Supplementary file1 (DOCX 41 KB)

## Data Availability

The climate data time series used in this paper is available on demand.

## Code availability

The numerical codes used for downscaling are available upon request.
