# Supplementary material for: Assessing the impacts of climate change on climatic extremes in the Congo River Basin
Source: Clim Change. 2022 Feb 28;170(3-4):40. doi: 10.1007/s10584-022-03326-x (PMC8885500; doi:10.1007/s10584-022-03326-x)
Supplement: Supplementary file 1 — Supplementary file1 (DOCX 41 KB) [file 10584_2022_3326_MOESM1_ESM.docx]

Or, in the case of the Congo, keys also include rehabilitating the

network of hydrometric stations and strengthening physiochemical laboratories.

Or, in the case of the Congo, keys also include rehabilitating the

network of hydrometric stations and strengthening physiochemical laboratories.

Or, in the case of the Congo, keys also include rehabilitating the

network of hydrometric stations and strengthening physiochemical laboratories.

Or, in the case of the Congo, keys also include rehabilitating the

network of hydrometric stations and strengthening physiochemical laboratories.

Appendix 1: Supplementary Material

For the evaporation estimation, the following Penman-Monteith equation derived for alfalfa was used:

$\lambda E=\frac{\Delta\cdot\left( H_{net}-G \right)+ \rho_{air}\cdot c_{p}\cdot[e_{z}^{0}- e_{z}]/r_{a}}{\Delta+\gamma\cdot(1+\frac{r_{c}}{r_{a}})}$ $r_{a}=\frac{114}{u_{z}}$

$$r_{c}=\frac{49}{1.4-0.4\frac{{CO}_{2}}{330}}$$

where λE = latent heat flux density (MJ m^-2^d^-1^);

E = depth rate evaporation;

Δ = slope of saturation vapor pressure-temperature curve (kPa ˚C^-1^);

H_net_ = the net radiation (MJ m^-2^d^-1^);

G = heat flux density to the ground (M.J. m^-2^d^-1^);

$\rho_{air}$= air density (kg/m^-3^)

c_p_ = specific heat at constant pressure (MJ kg^-1^ ˚C^-1^);

e_z_^0^ = saturation vapor pressure of air at height z (kPa);

e_z_ = water vapor pressure of air at height z (kPa);

ϒ = psychrometric constant (kPa ˚C^-1^);

r_c_ = plant canopy resistance (s/m);

r_a_ = aerodynamic resistance/diffusion resistance of the air layer (s/m).

${CO}_{2}$: concentration of carbon dioxide in part per millions (400)

Table S1 – Characteristics of the GCM/RCM model combinations used in the study

| No. | Driving GCM | GCM Institute | RCM | RCM Institute |
| --- | --- | --- | --- | --- |
| 1 | Canadian Earth System Model Version 2 | Canadian Centre for Climate Modeling and Analysis | Canadian Regional Climate Model 4 | Canadian Centre for Climate Modeling and Analysis |
| 2 | Canadian Earth System Model Version 2 | Canadian Centre for Climate Modeling and Analysis | Rossby Centre regional atmospheric model, version 4 | Swedish Meteorological and Hydrological Institute |
| 3 | Centre National de Recherches Météorologiques, Climate Model 5 | National Centre for Meteorological Research | Rossby Centre regional atmospheric model, version 4 | Swedish Meteorological and Hydrological Institute |
| 4 | Queensland Climate Change Centre of Excellence (QCCCE) and Commonwealth Scientific and Industrial Research Organization (CSIRO) | The Commonwealth Scientific and Industrial Research Organization | Rossby Centre regional atmospheric model, version 4 | Swedish Meteorological and Hydrological Institute |
| 5 | European community Earth-System Model | Irish Centre for High-End Computing | Rossby Centre regional atmospheric model, version 4 | Swedish Meteorological and Hydrological Institute |
| 6 | Institut Pierre Laplace Climate Model version 5A | Institute Pierre Simon Laplace | Rossby Centre regional atmospheric model, version 4 | Swedish Meteorological and Hydrological Institute |
| 7 | Model for Interdisciplinary Research on Climate, version 5 | Center for Climate System Research/ National Institute for Environmental Studies/ Frontier Research Center for Global Chance, Japan Agency for Marine-Earth Science and Technology | Rossby Centre regional atmospheric model, version 4 | Swedish Meteorological and Hydrological Institute |
| 8 | Hadley Centre Global Environment Model version 2 | Met Office Hadley Centre | Rossby Centre regional atmospheric model, version 4 | Swedish Meteorological and Hydrological Institute |
| 9 | Max Plank Institute Earth System Model | Max Planck Institute for Meteorology | Rossby Centre regional atmospheric model, version 4 | Swedish Meteorological and Hydrological Institute |
| 10 | Norwegian Earth System Model | Norwegian Climate Centre | Rossby Centre regional atmospheric model, version 4 | Swedish Meteorological and Hydrological Institute |
| 11 | Geophysical Fluid Research Laboratory Earth System Model | National Oceanic and Atmospheric Administration- Geophysical Fluid Dynamics Laboratory | Rossby Centre regional atmospheric model, version 4 | Swedish Meteorological and Hydrological Institute |
